# On the Origin of Testicular Germ Cell Tumors: From Gonocytes to Testicular Cancer

**DOI:** 10.3389/fendo.2019.00343

**Published:** 2019-06-06

**Authors:** Tiziano Baroni, Iva Arato, Francesca Mancuso, Riccardo Calafiore, Giovanni Luca

**Affiliations:** ^1^Department of Experimental Medicine, University of Perugia, Perugia, Italy; ^2^Department of Medicine, University of Perugia, Perugia, Italy; ^3^Division of Medical Andrology and Endocrinology of Reproduction, University of Perugia and Saint Mary Hospital, Terni, Italy

**Keywords:** germ cell neoplasia *in situ* (GCNIS), primordial germ cell (PGC), testicular germ cell tumors (TGCTs), testicular dysgenesis syndrome (TDS), Sertoli cells, Leydig cells

## Abstract

Human primordial germ cells (PGCs) have been described in the yolk sac wall around the beginning of the third week. From week 4 to 5, they migrate under control of SCF/c-KIT signaling pathway to the genital ridge, where they become gonocytes. PGCs and gonocytes express classic pluripotency markers, such as KIT, NANOG, and OCT3/4 that, during spermatogonia differentiation, are gradually suppressed, and substituted by the expression of some germ cell specific genes, such as VASA, SOX17, and TSPY. These genes, during normal development of germ cells, are tightly regulated by epigenetic modification, in terms of microRNA expression and DNA methylation. In adolescents and young adults, testicular germ cell tumors (TGCT) have a common precursor, the germ cell neoplasia *in situ* (GCNIS); the hypothesis of their origin from PGCs or gonocytes, whose maturation is altered, is widely accepted. The origin of TGCT, probably starting at early stages of embryogenesis, seems to be a part of the Testicular Dysgenesis Syndrome (TDS) where some early PGC/gonocytes, for still unclear reasons, are blocked in their differentiation, retaining their early marker profile. In this paper, current knowledge on the combination of epidemiological and genomic factors, involved in the development of testicular germ cell tumors, is reviewed.

## Introduction

The two main categories of testicular cancer fall into “germ cell,” representing up to 95% of testis malignancies, vs. “non-germ cell.” Tumors originated by germ cells are known as testicular germ cell tumors (TGCT) and can be divided into two main types: seminomas and non-seminomas according to their histological features. In about 10% of cases both seminoma and non-seminoma cells are present simultaneously in one testicle resulting in the so-called mixed germ tumors (1).

TGCT were extremely rare types of cancer until the second half of the twentieth century, when their prevalence arose dramatically and, for not yet elucidated reasons, have continued to steadily increase. In fact, the annual number of cases has more than doubled since the 1950s (2, 3).

TGCT, also known as Type II germ cell tumors (4), account for only 1% of all malignancies in males but in several Countries they are the most common solid tumors, occurring mainly in young men (18–35 years) (5) in which represent the leading cause of cancer-related death.

The incidence of this cancer shows geographic and ethnic differences: it is lowest (ranging from <0.5/100,000–5/100,000) in the majority of African and Asian Countries and highest (up to 12/100,000) in white populations of Northern European Countries. In particular, in the latter population, was observed, in 2012, significant differences in the incidence ranged from over 12/100,000 in Denmark and Norway to 5/100,000 in neighboring Finland or 5.4/100,000 in Italy and 3/100,000 in Spain (6).

Fortunately, the platinum-based chemotherapy has contributed to improve the mortality rate of TGCT worldwide from 1970 onwards and, today, the overall cure rate of TGCT is more than 90%; however about 10% of TGCT are unresponsive to chemotherapy, and 4–8% of relatively young patients, especially those with disseminated non-seminomas, die of the disease. These facts show the relevance to further improve our knowledge on the mechanisms underlying this disease.

**Figure 1:**
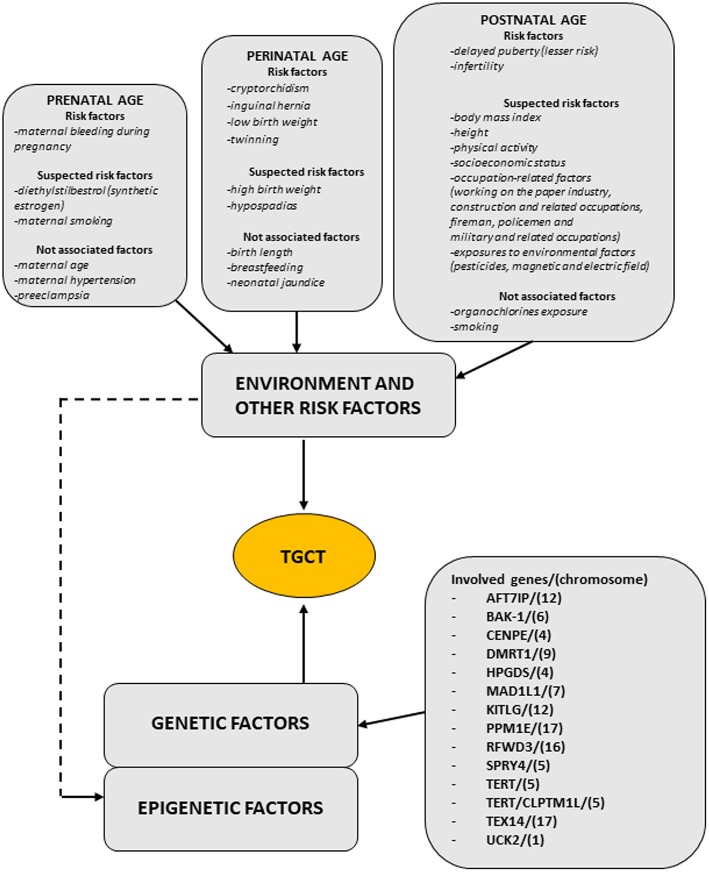
The scheme illustrates the genvironmental risk model showing different etiological factors influencing the development of TGCTs. Suspected risk factors and not associated factors are also listed. Modified from Elzinga-Tinke et al. (7).

Regarding TGCT etiopathogenesis, both inherited and environmental factors are thought to play a pivotal role, but at this time, there are insufficient evidences to make a risk assessment on any single individual factors. Figure 1 (modified by Asian J. Andr.) summarizes the “genvironmental hypothesis” that could, probably, explain the development of TGCT with a combined action of epigenetic and environmental factors (8).

## Risk Factor of TGCT

TGCT are considered the result of an altered germ cell differentiation that can be linked to the Testicular Dysgenesis Syndrome (TDS), a complex syndrome resulting from an abnormal fetal development of male gonads due to genetic, environmental factors or both (9, 10).

Various aspects of TDS (gonadal malformations, testicular microlithiasis, cryptorchidism, previous TGCT in the contralateral testis, disorders of sex development), altered fertility (subfertility/infertility), or hypospadias, are associated with increased risk of TGCT (9). For example, patients with previous history of TGCT have a relative risk of developing a contralateral malignancy about 25-fold higher than the age-matched general population (11).

Inherited genetic aberrations leading to disorders of sex development (DSD) are considered to affect gonadal development increasing the risk for GCNIS and TGCT. For example, 15–30% of patients 45XO/46XY DSD and 46XY DSD (with different degrees of gonadal dysgenesis) show the highest risk for TGCT (12).

Beyond karyotype, environmental factors may influence the risk for TGCT such as an excessive exposure to estrogen or molecules with estrogenic activity or endocrine disruptors during pregnancy (13).

Previous studies showed that environmental estrogens altered the normal development of embryonic urogenital system, resulting in an increase in cryptorchidism in newborns, and a decrease of total sperm counts associated with an increase in testis cancer rates in young men (14).

As demonstrated by other studies, mothers of patients with TGCT had higher estrogen levels during pregnancy (15) or were exposed to organic pollutants (16).

In this regard, it's fundamental to analyze the role of somatic cells. Indeed, also somatic Sertoli and Leydig cells, besides germ cells, could be affected in dysgenetic gonads. Their functions are to provide the appropriate microenvironment and the correct endocrine and paracrine signals for a normal germ cell development. So, altered testosterone levels could affect the normal development of somatic Sertoli cells (17) leading them to an insufficient germ cell stimulation and to an abnormal differentiation. In particular, a recent study performed on normal and neoplastic adult human testes led to the hypothesis that, in GCNIS tumors, Sertoli cell phenotype is changed to a less mature state (18).

Moreover, Sertoli cells secrete stromal cell-derived factor 1 (SDF1/CXCL12), a chemokine implicated both in PGC migration and regulation and support of adult stem cell niches. SDF1/CXCL12 binds to CXCR4 receptor located on both normal and TGCT cells and the signaling system lead to survival and growth of transformed cells thus facilitating the metastatic colonization of other organs.

Other important signaling systems expressed by Sertoli cells are represented by activin and inhibin, two members of the transforming growth factor beta (TGFbeta) superfamily that play a well-known role in spermatogenesis and FSH secretion.

Recently, it was demonstrated that activin A target genes are differentially expressed in neoplastic adult human testes compared to normal testes, thus suggesting a modulatory role of activin in the tumor niche and in TGCT development (18).

Inhibin B production is stimulated by androgens thus constituting a link between the microenvironment in where germ cells reside and the cells themselves. Importantly, inhibin is involved in the regulation of gonadal tumor development and progression (19).

In addition, endocrine disruptors may disturb regulatory actions exerted by androgens on somatic and germ cells (5). The latter could continue to express embryonic genes related to the undifferentiated state and pluripotency. Consequently, fetal gonocytes undergo abnormal cell division and accumulate chromosome aberrations facilitating their malignant transformation.

Some observations support the hypothesis of an involvement of sex hormone signaling. For example, TGCT develops only after puberty when the activated hypothalamic-pituitary-gonadal axis induces the transformation of GCNIS; in fact, patients affected by hypogonadotropic hypogonadism have a low risk of TGCT in cryptorchid testis (5).

Nevertheless, there is no evidence that the development of seminoma or non- seminoma TGCT are directly induced by sex hormones after birth. It seems more likely that hormones have an indirect effect when, during spermatogenesis, they promote GCNIS cell divisions leading to amplification of transformed cells bearing and accumulating various chromosome and genetic aberrations (20).

In summary, it is likely that imbalanced levels of maternal estrogens or environmental molecules with estrogenic activity during pregnancy could interact with specific genetic aberrations accumulated by GCNIS, playing a key role to promote tumorigenic pathology. In addition, affected Sertoli cells could create a defective microenvironment that allows arrested gonocytes to survive in the postnatal testes.

## From Gonocytes to Testicular Cancer

Previous studies about the origin of testicular cancers in adolescents and young adults (21, 22) demonstrated that TGCT have a common pathologic precursor, previously named carcinoma *in situ* (CIS) or Intratubular Germ Cell Neoplasia Unclassified (IGCNU) and, recently, according to an update of the 2016 World Health Organization classification (23), referred to as germ cell neoplasia *in situ* (GCNIS).

Further studies led to the currently most accepted hypothesis that GCNIS is an embryonic germ cell, that is a primordial germ cell (PGC) or a gonocyte, that failed to differentiate into a spermatogonium during development (24).

### Normal Germ Cell Development: From PGCs to Spermatogonia

Human PGCs have been described in the yolk sac wall during the 3–4 weeks post conception. From week 4 to 5, they migrate under control of SCF/c-KIT signaling system (25) in the hind gut epithelium and then they colonize the genital ridges, the precursors of both ovary and testis, where they are surrounded by supportive cells deriving from the coelomic epithelium. During and early after their migration PGC express specific markers, and some of these markers, such as OCT3/4, c-KIT, placenta like alkaline phosphatase (PLAP) and NANOG could be used as diagnostic markers for TGCT and GCNIS (26).

At 6th week, the expression of SRY gene in the male embryo lead to differentiation of genital ridges into testes (27) inducing the expression of SOX9, a transcription factor that initiates the differentiation of supportive cells into Sertoli cells (28). Sertoli cells organize the microenvironmental niche regulating germ cell differentiation into spermatogonia until the first month after birth, when the mitotic arrest takes place (29).

**Figure 2:**
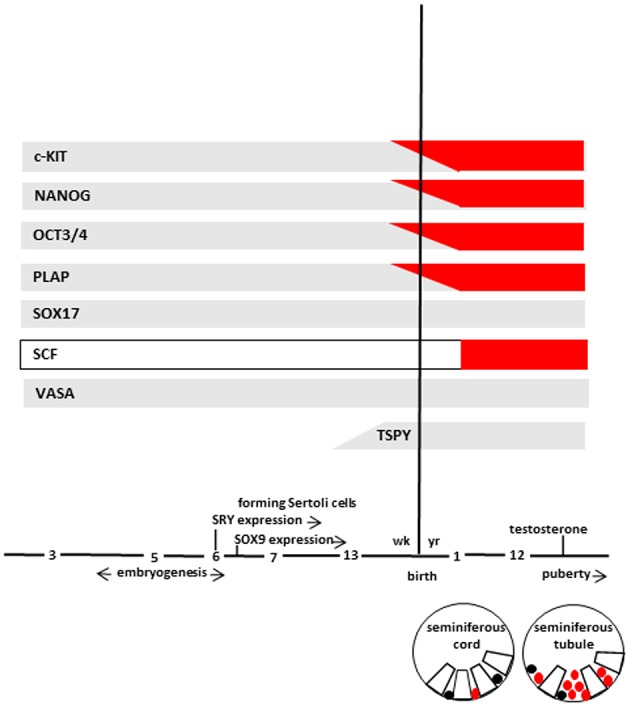
The scheme represents marker expression from early embryogenesis to puberty during the normal (gray bar) and impaired (red bar) testicular development leading to GCNIS. Germ cells (in different stages of maturation), and GCNIS cells are represented as black or red circles, respectively. Sertoli cells are represented as open white boxes. Modified from Elzinga-Tinke et al. (7).

At 7th week, primitive seminiferous cords, a particular structure in which germ cells and Sertoli cells are not yet organized, are formed. Subsequently, germ cells migrate toward the basal lamina of the seminiferous cords (if not migrate they undergo to apoptosis and cleared from the seminiferous epithelium) and, during the 13th week, germ cells start to lose the expression of some markers (c-KIT, OCT3/4, and PLAP). In particular, c-KIT can still be detected at a relatively low level, while OCT3/4 and PLAP disappear completely. On the contrary, VASA and SOX17 continue to be expressed remaining positive throughout life (Figure 2). In addition, at same time, gonocytes express TSPY, which regulates the normal proliferation of spermatogonia and remain positive up to meiotic division.

PGCs differentiation passes through three stages which three different types of germ cells: gonocytes, intermediate cells, and spermatogonia, concurrently present in the fetal testis and distinguishable by morphologic and immunohistochemical features (30).

In particular, gonocytes are large cells with spherical euchromatic nuclei with one or two nucleoli (31). At the 10th week of gestation, they are the more abundant type of germ cells located centrally within the seminiferous cords and separated from the basal lamina by Sertoli cells. Then, gonocytes become intermediate cells, with similar morphology but located peripherally within the seminiferous cords and in contact with the basal lamina. At gestational week 15, many intermediate cells are present together with gonocytes. It has been hypothesized that when these cells reach the basal lamina, they lose their pluripotency and start to differentiate into spermatogonia. From the 18th week onward, spermatogonia constitute the most common germ cell population. They are located peripherally to the basal lamina and enter mitotic arrest.

With regards to molecular features the three different types of germ cells populations (gonocytes, intermediate cells and spermagonia) express different markers of pluripotency (Table 1) and show different epigenetic modifications.

**Table 1:** The table summarizes the expression of different markers in the three different stages of germ cell differentiation from PGC to spermatogonium.

**Germ cell type**	**Marker**
Gonocyte	OCT3/4 NANOG c-KIT PLAP
intermediate cell	OCT3/4 low positive or negative PCNA
Spermatogonium	MAGE4A VASA TSPY

In particular, gonocytes express markers of pluripotency (OCT3/4, NANOG, and c-Kit), and are positive for placental alkaline phosphatase (PLAP). Normally, gonocytes are negative for melanoma-associated antigen 4 (MAGEA4); in fact this marker is expressed in the fetal germ cells from 17 weeks of gestation onward (30).

Intermediate cells are negative for both c-KIT and MAGEA4, and show low or negative staining for OCT3/4 and positive staining for proliferating cell nuclear antigen (PCNA), marker of proliferative activity. Beginning from late gestation (week 17–18) until about 1 year of post-natal life, spermatogonia loss fetal markers (the cells are negative for c-KIT and PCNA) and start the expression of germ cell specific markers such as MAGE4A, VASA, and testis-specific protein Y-encoded (TSPY) gene (32–34).

Regarding the epigenetic modifications, genes involved in germ cell development are strictly regulated by epigenetic changes, such as DNA methylation, and microRNA (miRNA) activity (see below) (35, 36).

In particular, gonocytes show loss of genomic methylation. *De novo* DNA methylation will start in spermatogonia to re-establish the parental imprinting pattern (35).

The maturation processes that lead from gonocyte to spermatogonia are not synchronized and therefore in seminiferous tubules are present germ cell populations expressing embryo/fetal markers, differentiation markers, methylated or un-methylated genes.

### From Normal Germ Cells to GCNIS

The GCNIS cells, located above the basal lamina, show abundant cytoplasm and large spherical or irregular nucleus with tetraploid DNA content with 1 or 2 nucleoli (37). These cells are present in 0.4–0.8% of men among where spermatogenesis is reduced or absent. Their presence is hardly diagnosed because of the absence of symptoms. Generally, it is estimated that 70% of GCNIC-positive male subjects will develop TGCT within 7 years (38) with a median age at cancer diagnosis of 35 years.

The most widely accepted hypothesis about GCNIS origin states that they are germ cells in which an arrest of the development has occurred for an abnormal signals or inability to respond to correct signals. The cells continue to express their pluripotency markers, do not differentiate and remain quiescent until puberty. In the quiescent period, GCNIS cells could accumulate chromosomal aberrations that affect genes involved in proliferation and differentiation that lead them to an uncontrolled and potentially malignant growth (39) in coincidence with puberty, when growth signals and hormones produced by Sertoli and Leydig cells induce GCNIS to proliferate.

Previous studies on chromosomal aberrations in invasive seminoma and non- seminoma neoplasms demonstrated that 80–100% of these tumors and GCNIS cells adjacent to cancer exhibited a gain of the short arm of chromosome 12 (or smaller parts thereof) (40) usually in the form of an isochromosome, i(12p) chromosome (41).

This event suggests that gain of 12p could play a key role for TGCTs to acquire invasive ability given that GCNIS cells, that are relatively distant from the cancerous zone, normally do not present short arm of chromosome 12 gain.

In fact, the chromosomal region corresponding to 12p contains genes that could be associated to TGCT development, such as NANOG, STELLA, GDF3, and Cyclin D2 (CCND2). In particular, NANOG, STELLA, and GDF3 are pluripotency-related genes, and play an important role in embryonic stem cell self-renewal, whereas CCND2 is involved in the cell cycle regulation. These genes could similarly induce pluripotency in GCNIS (42).

Histologic and biomolecular studies demonstrated several likeness among TGCT and their precursor GCNIS cells. For example, pluripotency markers such as OCT3/4 and NANOG (43–45) are expressed in a similar way by PGCs, fetal gonocytes and GCNIS. In addition, GCNIS cells exhibit several features of PGCs and gonocytes such as the co-expression of OCT3/4 and SOX17 protein (46, 47). Moreover, high c-KIT gene expression was detected in GCNIS similarly to PGCs and fetal gonocytes but not in the adult spermatogonia (43). Similarly, an upregulated c-KIT expression was described in atypical fetal gonads thus strengthening the idea that germ cell transformation and altered testicular development might be strictly associated (48) (Figure 2).

Interestingly, GCNIS cells share mRNA/miRNA profiles similar to immature germ cells, and exhibit global CpG methylation erasure. This lack of epigenetic memory is a common feature of PGCs and pluripotent cell types (49).

However, even though PGCs express various biomarkers of pluripotency, they are normally unipotent to produce gametogenic stem cells, so differing from GCNIS cells that exhibit pathologic functional pluripotency.

Taken together, all these findings have led to the hypothesis that GCNIS is the intermediary cell between an arrested and transformed PGC or gonocyte during embryonic/fetal development and TGCT (50).

### Future Perspectives About Diagnostic Markers

Diagnosis for TGCTs is greatly based on detecting serum markers such as alfa- fetoprotein, beta-human chorionic gonadotropin, and lactate dehydrogenase but only 60% of all patients show elevations of these markers (51).

Testicular biopsy is the best current diagnostic test for detecting TGCT, even if it is burdened with false negative outcomes due to the non-random distribution of transformed cells throughout the gonad (52). New approaches are necessary to identify GCNIS before testicular cancer appearance, given that these cells can leave the testis and enter the semen where they could be detected by revealing specific markers. However, for some of the assayed markers as OCT3/4, MAGE-A4, and NY- ESO-1 a relatively low sensitivity was demonstrated (53–55).

Recently, a cell surface receptor TDGF-1 (CRIPTO) was identified in blood serum of patients where GCNIS and several tumor cell subtypes were found (56). Therefore, CRIPTO expression could be a useful serum marker for detection of testicular cancer.

Other recent studies showed that undifferentiated and potentially malignant cells could be detected *in vivo* thanks to identification of specific miRNAs (57).

In particular, miRNAs from miR-371–373 (mapped to chromosome 19) and miR- 302–367 (mapped to chromosome 4) family members are upregulated in all TGCT and elevated values could be detected in the serum, regardless of pediatric or adult age, gonadal or extragonadal localization or tumor subtype (seminomas, yolk sac tumors, or embryonal carcinomas) (58).

It's noteworthy that these miRNAs are not up-regulated in other tumor types or disorders. In perspective, detection of high levels in liquid biopsies of well-defined set of embryonic miRNA, such the **two** above mentioned “clusters,” might be useful in diagnosis, prognosis and disease management of testicular malignant TGCTs given their association with undifferentiated and potentially malignant cells (59).

A more recent study based on microarray gene expression profiling and gene methylation datasets, suggests that hypomethylation-high expressed genes such as CSF1R, PTPRC, and MMP9, could be involved in TGCT (60).

CSF1R, a cell-surface protein, and PTPRC, a member of the protein tyrosine phosphatase (PTP) family, regulate several cellular activities such as cell growth, differentiation, and tumor transformation (61).

Moreover, this study demonstrates that TGCT tissue samples show up-regulated levels of MMP9, a class of enzymes involved in the degradation of the extracellular matrix.

About this, another recent study shows that activin/TGFbeta signaling within Sertoli cells of GCNIS tumors lead to increased levels of MMP2 and MMP9 metalloproteinases (18) thus strengthening the idea that Sertoli cells have an important role in supporting TGCT development. Indeed, the breakdown of the epithelial barrier by MMPs may contribute to tumor progression, thus allowing the neoplastic germ cell to move into the interstitium.

Overall, seems that higher levels of CSF1R, PTPRC, and MMP9 are related to shorter survival time of TGCT patients, suggesting that they may be involved in TGCT development.

In perspective, these genes could be useful biomarkers for diagnosis, treatment and prognosis evaluation of TGCT, constituting potential therapeutic targets for this type of cancer.

Finally, we must not overlook the fact that dysregulation between somatic and germ cells may support the formation of GCNIS cells, as demonstrated by the role of activin/TGFbeta signaling in promoting an environment advantageous for TGCT onset and progression.

## Conclusions

Testicular cancer onset and development is caused by a mix of genetic, epigenetic and environmental factors. Most TGCT tumors are curable even in advanced stages thanks to cisplatin-based chemotherapy. However, side effects and complications may occur in patients treated with chemotherapeutic agents and in some cases relapse or treatment resistance may occur.

Further studies will be aimed to both develop less toxic therapies and directly target the neoplastic cells, thus overcoming the resistance to chemotherapy.

Currently, an open testicular biopsy, helpful in specific group of patients (men with atrophic testes, infertility, cryptorchidism or suspicious ultrasound), is the sole way to diagnose GCNIS as other early detection methods for TGCT are not available so far.

Obviously, for screening purposes, sensitive and specific non-invasive early detection method are necessary.

Even if genetic and environmental factor of risk (prenatal, perinatal, and postnatal) were considered able to influence the onset of GCNIS, their role in the pathogenesis of TGCT is insufficient to identify an at risk population.

Even if many cytoplasmic and nucleus markers (such as OCT3/4, NANOG, etc.) have been assessed in semen, none of these is a valid marker for GCNIS.

Instead, the detection of specific TGCT's miRNAs (miR-371~373 and miR-302/367) in semen could be considered a promising non-invasive marker of GCNIS being highly overexpressed both in serum (in all TGCT) and in semen. In addition, MMP9, CSF1R, and PTPRC genes could be useful biomarkers for diagnosis, treatment and prognosis evaluation of TGCT.

In conclusion, improving our knowledge on the molecular mechanisms controlling GCNIS origin and malignant transformation to TGCT, might be useful to develop a noninvasive screening method for population at increased risk for TGCT.

## Author Contributions

TB, IA, and GL idealized the paper and wrote the first draft. FM and RC participated in literature research and paper writing. All author listed have made intellectual contribution to the work and approved the final version.

### Conflict of Interest Statement

The authors declare that the research was conducted in the absence of any commercial or financial relationships that could be construed as a potential conflict of interest.
